# Prophylactic orthosteric inhibition of leukocyte integrin CD11b/CD18 prevents long-term fibrotic kidney failure in cynomolgus monkeys

**DOI:** 10.1038/ncomms13899

**Published:** 2017-01-10

**Authors:** Abbas Dehnadi, A. Benedict Cosimi, Rex Neal Smith, Xiangen Li, José L. Alonso, Terry K. Means, M. Amin Arnaout

**Affiliations:** 1 Division of Transplant Surgery, Department of Surgery, Massachusetts General Hospital, Boston, Massachusetts 02114, USA; 2 Harvard Medical School, Boston, Massachusetts 02115, USA; 3 Department of Pathology, Massachusetts General Hospital, Boston, Massachusetts 02114, USA; 4 Leukocyte Biology and Inflammation Program, Massachusetts General Hospital, Boston, Massachusetts 02114, USA; 5 Division of Nephrology, Department of Medicine, Massachusetts General Hospital, Boston, Massachusetts 02114, USA; 6 Division of Rheumatology, Department of Medicine, Massachusetts General Hospital, Boston, Massachusetts 02114, USA; 7 Center For Regenerative Medicine, Medical Services, Massachusetts General Hospital, Boston, Massachusetts 02114, USA

## Abstract

Ischaemic acute kidney injury (AKI), an inflammatory disease process, often progresses to chronic kidney disease (CKD), with no available effective prophylaxis. This is in part due to lack of clinically relevant CKD models in non-human primates. Here we demonstrate that inhibition of the archetypal innate immune receptor CD11b/CD18 prevents progression of AKI to CKD in cynomolgus monkeys. Severe ischaemia-reperfusion injury of the right kidney, with subsequent periods of the left ureter ligation, causes irreversible right kidney failure 3, 6 or 9 months after AKI. Moreover, prophylactic inactivation of CD11b/CD18, using the orthosteric CD11b/CD18 inhibitor mAb107, improves microvascular perfusion and histopathology, reduces intrarenal pro-inflammatory mediators and salvages kidney function long term. These studies reveal an important early role of CD11b^+^ leukocytes in post-ischaemic kidney fibrosis and failure, and suggest a potential early therapeutic intervention to mitigate progression of ischaemic AKI to CKD in humans.

## Abstract (web summary)

Acute kidney injury can progress to chronic kidney disease. Here Dehnadi *et al*. develop a post-ischaemic chronic kidney disease model in cynomolgus monkeys and show that prophylactic inhibition of CD11b/CD18 leukocyte receptor via a monoclonal antibody inhibits progression of kidney disease and fibrosis.

Acute kidney injury (AKI), often caused by ischaemia-reperfusion (I/R)^1^, is a common and growing burden for the healthcare system worldwide^2^. AKI is a strong predictor of chronic fibrosis and end-stage renal disease in humans, especially following severe (dialysis-requiring) injury to native or transplanted kidneys^3^,^4^. Whether AKI per se or the associated comorbidities is the cause of the progression to chronic kidney disease (CKD) remains controversial^5^,^6^. In addition, despite important advances in understanding the pathogenesis of AKI, no effective prophylaxis currently exists.

Acute kidney ischaemia induces release of inflammatory mediators that activate resident mononuclear phagocytes (macrophages and dendritic cells), the majority (>90%) of which express integrin CD11b^7^. After reperfusion, inflammation becomes self-sustaining with recruitment of CD11b^+^ blood leukocytes, which produce further cytotoxic (free radical species, lipid mediators and proteases) and proinflammatory mediators (cytokines, chemokines and complement^8^). When the acute injury is mild, it may be followed by a reparative phase, with restoration of normal tissue homeostasis, but progressive scarring often follows severe injury^9^. Mononuclear phagocyte subsets are involved in both the reparative and fibrosis phases of injury^10^ and often switch roles in repair and fibrosis^11^, complicating target selection and timing of therapeutic interventions. Translational studies targeting an array of proinflammatory mediators have identified several apparently efficacious agents primarily in rodent models of AKI; however, these failed to improve recovery or halt progression to CKD in humans^12^,^13^. Contributory factors probably include the lack of reliable models in non-human primates, given the known differences in immune responses between rodents and humans^14^, the relatively short follow up period, which may not reflect the long-term changes in the injured kidneys^15^, and the multiplicity of proinflammatory mediators and their complex roles in tissue injury and repair.

In this study, we developed a rigorous CKD model in healthy outbred cynomolgus monkeys and used it to evaluate the role of CD11b-expressing immune cells in progression of I/R AKI to CKD. CD11b pairs with CD18 to form a heterodimeric integrin receptor expressed on resident and circulating phagocytes, fibrocytes, natural killer (NK) cells and some mast cells, CD8^+^ and γδ T cells^10^,^16^,^17^,^18^,^19^,^20^. CD11b/CD18 binds more than 30 ligands including iC3b, fibrinogen, intercellular adhesion molecule 1 (ICAM-1) and double-stranded RNA, and mediates phagocytosis, antibody-dependent cellular cytotoxicity, adhesion-dependent release of proinflammatory mediators, and phagocyte–platelet-, phagocyte–endothelial cell- and phagocyte–epithelial cell adhesion^16^,^21^. CD11b/CD18 also acts as a signalling partner for TLR4 and Fcγ receptors IIa and III^22^,^23^, which have been shown to be pathogenic in AKI models^24^,^25^. We have generated a mouse mAb107 that inhibits binding of CD11b/CD18 to multiple ligands^26^ and subsequently showed that it is a ligand mimetic that binds the ligand-binding site in the CD11b αA domain, but does not inadvertently activate the receptor^27^. The latter unique feature contrasts with current orthosteric anti-integrin drugs that act as partial agonists, a property linked to adverse outcomes in patients^28^. We now show that inactivation of CD11b/CD18 by prophylactic administration of mAb107 at the onset of I/R AKI ameliorated progression to fibrotic CKD and explore the underlying mechanisms.

## Results

### A non-human primate model of CKD

**Figure 1:**
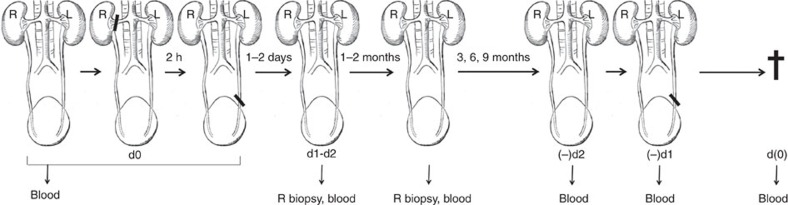
Schematic of the protocol used to develop the CKD model in monkeys. See text for details. d0, day of surgery; d1 and d2, 1 and 2 days after injury. (−) d2 and (−) d1, 2 and 1 day before planned euthanasia; d(0) immediately before euthanasia; L, left; R, right; †, euthanized.

We modified an earlier model of ischaemic AKI in non-human primates^29^, to assess the long-term impact of severe AKI on progressive kidney fibrosis and failure (Fig. 1). In a pilot study of two cynomolgus monkeys, we induced unilateral warm ischaemic AKI by a 2 h *in situ* vascular occlusion of the right kidney, with ligation of the contralateral left ureter. This protocol led to acute rise in serum creatinine (Cr), necessitating release of the left ureter ligature 2 days later to avoid animal death from uremia. Histopathology of the right kidneys harvested 3 months after AKI showed widespread tubular atrophy and tubulointerstitial fibrosis. In the present studies, the right kidney function was also measured at 3, 6 or 9 months after AKI by religating the left ureter 2 days before euthanizing the animal, to test residual right kidney function (Fig. 1).

### Effect of mAb107 on unilateral ischaemic AKI

**Figure 2:**
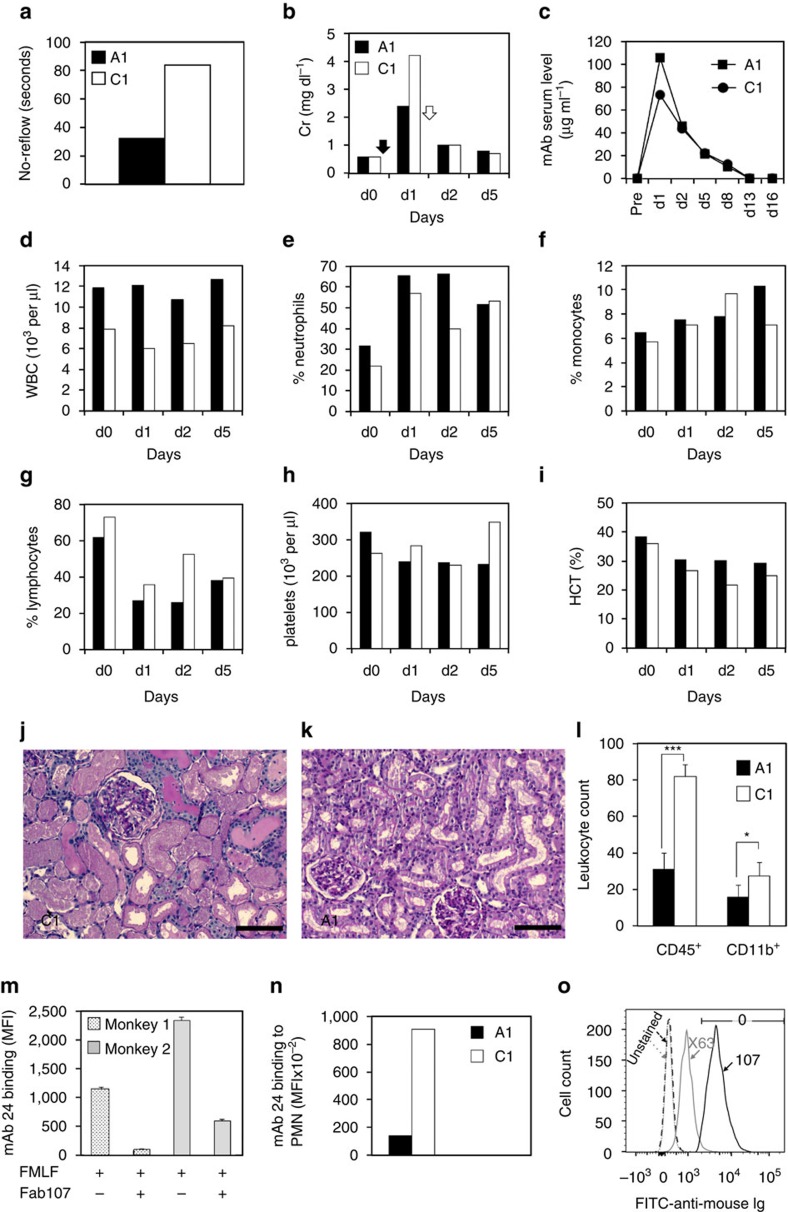
Acute right kidney injury in a treated and a control monkey. One monkey received mAb107 (A1, filled histogram) and the other the isotype-matched mAbX63 (C1, open histogram). (**a**) The no-reflow period. (**b**) Serum creatinine (Cr) before surgery (d0) and vascular clamp application (filled arrow), and on d1 after injury. After release of left ureter ligature (open arrow), Cr returned to normal by day 5 after AKI. (**c**) Serum levels of mAb107 and mAbX63 in A1 and C1, respectively, 1–16 days after AKI. (**d**–**i**) White blood cell (WBC), neutrophil, monocyte, lymphocyte and platelet counts and hematocrit (HCT) measured on d0, and on d1, d2 and d5 after injury in mAb107-treated (filled histogram) or control (open histogram). (**j**,**k**) Representative Periodic Acid Schiff (PAS)-stained right kidney sections from C1 (**j**) and A1 (**k**) biopsies taken 1 day after AKI, showing marked tubular necrosis in C1 as compared with less severe tubular injury in A1. Scale bars, 200 μm (**j**,**k**). (**l**) Histograms (mean+s.d.) showing numbers of intrarenal CD45^+^ and CD11b^+^ cells (five random fields at × 20 magnification were examined). ***P<0.0001 and *P<0.05. (**m**) Histograms (mean+s.e.) of triplicate determinations showing binding of mAb24 to chemoattractant (f-met-leu-phe (FMLF))-activated PMN from two naive monkeys in the absence or presence of the Fab fragment of mAb107. MFI, mean fluorescence intensity. (**n**) Histograms showing binding of mAb24 to PMN isolated from A1 and C1 monkeys 1 day after injury, when serum levels of mAb107 and X63 were at a peak. (**o**) Flow cytometric analysis of the PMN studied in **n**, unstained or stained with fluorescein isothiocyanate (FITC)-labelled anti-mouse Ig. *P-*values in **l** were calculated using the Mann–Whitney *U*-test.

The above-modified protocol was tested in a pair of healthy cynomolgus monkeys. One animal (A1, 4.2 kg) received intravenous mAb107 at 6 mg kg^−1^, with half the dose given 2 min before ischaemia and the other half immediately before reperfusion. The control animal (C1, 3.0 kg) received an equal dose of an IgG1-matched non-reactive mouse mAbX63 (American Type Culture Collection, Rockville, Maryland). The surgical team was blinded as to which animal received the respective monoclonal antibody. On release of the right vascular obstruction, no-reflow time, which reflects microvascular perfusion^30^,^31^, was nearly threefold longer in C1 versus A1 (Fig. 2a and Supplementary Movies). Cr rose by four- and sevenfold in A1 and C1, respectively, 1 day after I/R (Fig. 2b). Release of the left ureteral ligature normalized Cr in both animals by day 5 (Fig. 2b). One day after AKI, serum levels of mAb107 and mAbX63 were 105.6 and 73.8 μg ml^−1^, respectively, and progressively declined, becoming undetectable by day 13 (Fig. 2c). mAb107 did not deplete circulating red or white blood cell subsets or platelets on days 1, 2 and 5 post injury (Fig. 2d-i). A treatment-blinded histopathology examination of right kidney biopsy 1 day after injury revealed extensive acute tubular injury and necrosis in the control but preservation of tubular architecture in the treated animal (Fig. 2j,k). CD45^+^ and CD11b^+^ leukocytes were significantly reduced at this time in treated versus control right kidneys (Fig. 2l).

### mAb107 inhibits integrin activation

As in human polymorphonuclear cells (PMN)^27^, binding of mAb107 to PMN isolated from two naive monkeys blocked the chemoattractant f-met-leu-phe-induced conversion of monkey CD11b/CD18 to the high-affinity proadhesive state, which was detected by binding of the activation-sensitive mAb24 (ref. 32 and Fig. 2m). Flow cytometric analysis of PMN obtained 1 day after AKI showed a remarkable reduction in binding of APC-mAb24 to PMN from the mAb107-treated versus control animal (Fig. 2n). Binding of mAb107 to PMN isolated from the treated monkey was confirmed by the selective binding of fluorescein isothiocyanate-labelled goat anti-mouse Ig to these cells (Fig. 2o). Thus, early administration of mAb107 prevents the activating conformational switch of monkey CD11b/CD18 *in vivo*, despite the presence of high serum levels of the known CD11b/CD18 agonists interleukin (IL)-8 and monocyte chemoattractant protein-1 (MCP-1) in the mAb107-treated animal (Supplementary Fig. 1).

### Post-ischaemic kidney function in monkeys A1 and C1

**Figure 3:**
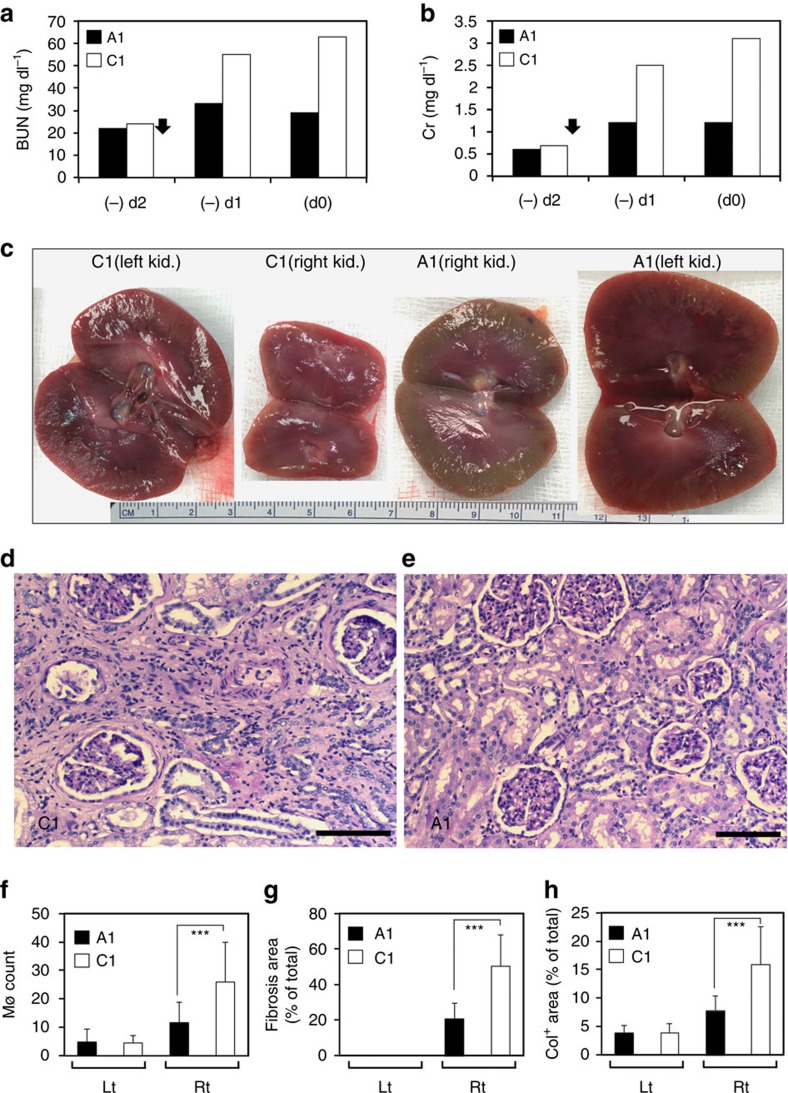
Progression of AKI to CKD in a treated and a control monkey. (**a**,**b**) BUN and Cr levels on (−)d2 and (−)d1, and d(0). Closed arrow, left ureter religation. (**c**) Gross appearance of harvested left and right kidneys from C1 and A1. (**d**,**e**) Periodic Acid Schiff (PAS)-stained sections of the control (C1) and treated (A1) right kidneys harvested at 3 months after ischaemic AKI. Tubular atrophy and interstitial fibrosis are seen in **d** (C1) compared with mild changes seen in **e** (A1). Scale bars, 200 μm (**d**,**e**). (**f**) Histograms (mean+s.d.) showing macrophage (Mø) counts in 20 high-power (× 200) fields. ***P<0.0001. (**g**) Histograms (mean+s.d.) showing the fibrotic area (trichrome stain-positive) presented as % of total area in twenty random fields at × 20 magnification. ***P<0.0001. (**h**) Histograms (mean+s.d.) showing collagen staining expressed as percent of pixels positive for anti-Col antibody in 20 random fields at × 40 magnification. ***P<0.0001. *P*-values in **f**–**h** were calculated using the Mann–Whitney *U*-test.

Because of the potential bias in biopsy tissue sampling, the contralateral left ureter was religated 3 months after AKI, allowing serial assessments of the right kidney function 1 and 2 days later. Blood levels of urea nitrogen (BUN) and Cr rapidly rose in C1 from respective baselines of 22/0.7 mg dl^−1^ to 55/2.5 (day 1) and 63/3.1 (day 2; Fig. 3a,b). In contrast, BUN/Cr in the treated animal rose from baselines of 22/0.6 only to 33/1.2 (day 1) and 29/1.2 (day 2; Fig. 3a,b). Both animals were euthanized on day 2 and kidneys were harvested, visually examined and submitted for treatment-blinded histopathology. The control right kidney was adherent to the surrounding tissue and was visibly smaller than the control left kidney, with marked thinning of the outer cortex (Fig. 3c). In contrast, the mAb107-treated right kidney did not adhere to the surrounding tissue and was only slightly smaller than the same animal's left kidney (Fig. 3c). Histopathology of the left kidney from both animals showed the expected acute tubular injury secondary to the 2-day pre-euthanasia left ureteral obstruction. Extensive injury and fibrosis was observed in the control right kidney (Fig. 3d), with significant recovery of tubular architecture in the treated kidney (Fig. 3e). Intrarenal macrophages, interstitial fibrosis and collagen deposition were all significantly reduced in the treated right kidney, but did not drop to the baseline level seen in the left kidney (Fig. 3f–h).

### Kidney function in four additional pairs of monkeys

**Figure 4:**
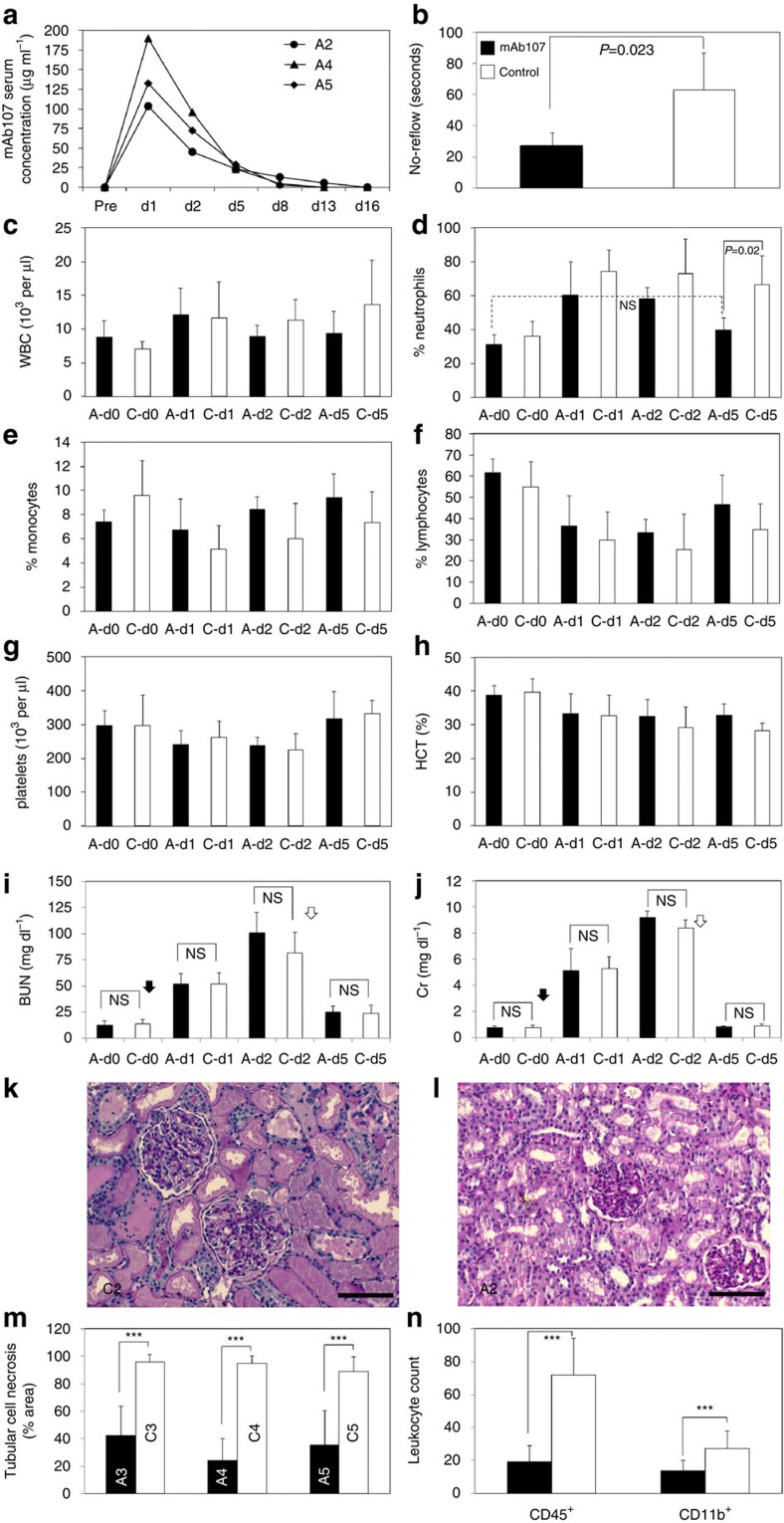
Progression of AKI to CKD in two groups of monkeys. (**a**) Serum levels of mAb107 in treated monkeys (A2, A4 and A5), before and up to 16 days after kidney injury. (**b**) Histograms (mean+s.d., *n*=4) showing duration of no-reflow in mAb107-treated and control monkeys. In this and subsequent figures, filled and open histograms represent the mAb107-treated and control groups, respectively. (**c**–**h**) White blood cell (WBC), neutrophil, monocyte, lymphocyte, platelet and hematocrit (HCT) levels in treated (A) and controls (C), measured on d0 and on d1, d2 and d5 after injury. (**i**,**j**) BUN/Cr levels on d0 and on d1, d2 and d5 after injury (filled and open arrows, indicate times of left ureter clamping and release, respectively). Data in **b**–**j** are shown as histograms (mean+s.d.) and were analysed by the Student *t*-test. NS, not significant. (**k**,**l**) Representative Periodic Acid Schiff (PAS)-stained right kidney sections from C2 (**k**) and A2 (**l**) biopsies taken 2 days after injury, showing marked tubular necrosis in C2 as compared with less severe tubular injury in A2. Scale bars, 200 μm (**k**,**l**). (**m**) Histograms (mean+s.d.) showing tubular epithelial cell necrosis in right kidney biopsies 2 days post injury from the three remaining monkey pairs. Ten random fields at × 20 magnification were examined. ***P<0.0001. (**n**) Histograms (mean+s.d.) showing number of intrarenal CD45^+^ and CD11b^+^ cells in respective 30 and 25 random fields (× 20 magnification), of 2-day right kidney biopsies. ***P<0.0001. *P*-values in **m**, **n** were calculated using the Mann–Whitney *U*-test.

To assess the significance of the above findings in a larger cohort and the longer-term effects of preemptive mAb107 treatment on the right kidney function following I/R, the above studies were repeated in eight additional monkeys, randomized to four in each group, a number expected to yield highly significant (*P*≤0.2) differences based on the initial pair. We also extended the follow-up period to 6 months after injury in two pairs (A2/C2 and A3/C3) and to 9 months in the two others (A4/C4 and A5/C5). Serum levels of mAb107 measured in three of the four treated animals yielded values comparable to those seen in A1 and followed similar pharmacokinetics (Fig. 4a).

No-reflow was shortened significantly in the treated versus control group (Fig. 4b). mAb107 did not deplete circulating red or white blood cell subsets, or platelets 1 and 2 days after administration (Fig. 4c–h). Neutrophil counts returned to baseline levels on day 5 in the treated group, but remained elevated in the control group (Fig. 4d), a probable reflection of persistent systemic inflammation. BUN/Cr rose to equivalent levels in the two groups 1 and 2 days after the left ureter ligation (Fig. 4i,j) and returned to normal by day 5 after release of the left ureter ligature (Fig. 4i,j). Right kidney biopsies showed widespread tubular necrosis (Fig. 4k,m) and higher numbers of CD45^+^ and CD11b^+^ leukocytes in the control group (Fig. 4n) versus the treated animals (Fig. 4l–n).

### Kidney function and histopathology 6–9 months after AKI

**Figure 5:**
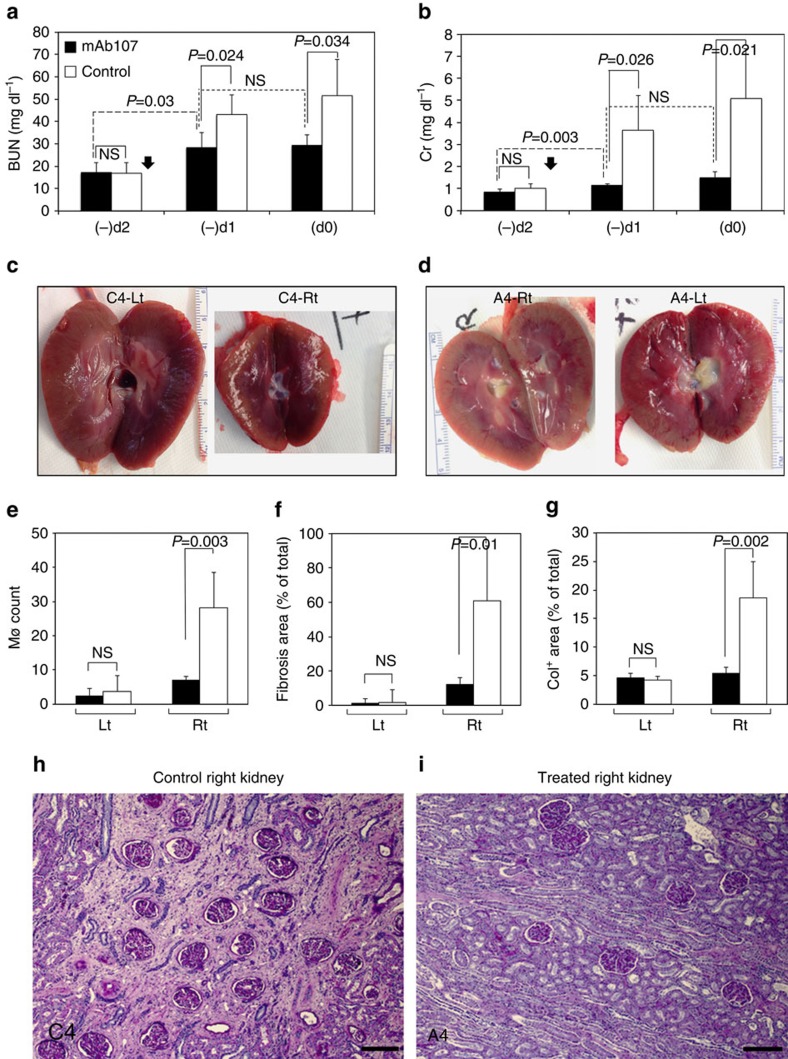
mAb107 salvaged right kidney function several months after AKI. (**a**,**b**) BUN/Cr levels 2 days (−d2), one day (−d1) and (d0) before planned euthanasia (mean+s.d.; filled arrow, time of left ureter religation). (**c**,**d**) Representative gross appearance of left (Lt) and right (Rt) kidneys from control (C4) and treated (A4) monkeys harvested 9 months after AKI. (**e**–**g**) Histograms (mean+s.d.) showing number of macrophages (Mø) (**e**), fibrosis (**f**) and collagen deposition (**g**) in Lt and Rt kidney sections. Data in **a**, **b**, **e**–**g** were analysed by the Student *t*-test. NS, not significant. (**h**,**i**) Representative Periodic Acid Schiff (PAS)-stained sections of the control (C4) and treated (A4) right kidneys shown in **c** and **d**. End- stage tubular atrophy and interstitial fibrosis are seen in **h**, compared with the mild changes seen in **i**. Scale bars, 200 μm (**h**,**i**).

The right kidney function was terminally measured 1 and 2 days following religation of the contralateral left ureter in seven of the eight animals 6 and 9 months after AKI (three treated and four controls). BUN/Cr levels were significantly lower in the mAb-107-treated group 1 day after the left ureter religation versus controls and did not rise further on day 2 (Fig. 5a,b).

Harvested control right kidneys were again more adherent to surrounding tissue and had abnormal tissue architecture when compared with their left counterparts or to the right kidneys from mAb107-treated animals (representative images from A4/C4 are shown in Fig. 5c,d). Significant increases in interstitial macrophages, tubulointerstitial fibrosis and collagen deposition were also observed in all four controls (Fig. 5e–g). Histopathology of kidneys from each of the mAb107-treated animals showed tubular recovery, with minimal or only focal areas of fibrosis (Fig. 5h,i, images of the right kidneys of A4/C4 harvested at 9 months after injury).

### Inflammatory mediator profiles in animals following injury

**Figure 6:**
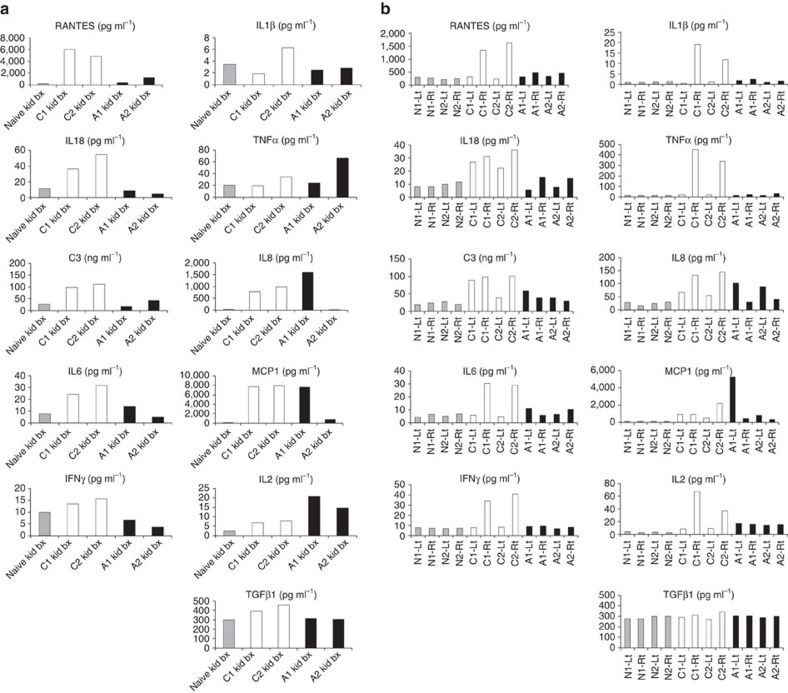
Effect of mAb107 on proinflammatory mediators in kidney tissue. (**a**,**b**) Cytokine/chemokine and complement C3 levels in right kidney biopsies taken 1–2 days after AKI (**a**), and in the left and right kidneys harvested 3 (C1, A1) and 9 (C2, A2) months after AKI (**b**). A naive kidney biopsy (**a**) and four harvested naive (N) kidneys (**b**) (shaded histograms) served as controls. All values are expressed as amount of inflammatory mediator per μg kidney tissue.

A number of chemokines, cytokines and complement were found to be pathogenic in rodent models of ischaemic AKI^8^. To assess the impact of mAb107 on production of inflammatory mediators, we quantified the protein levels of selected proinflammatory mediators in the right kidney biopsies taken 1–2 days after AKI from two mAb107-treated (A1 and A2) and two control (C1 and C2) animals. Levels of regulated on activation, normal T cell expressed and secreted (RANTES), IL-18, complement C3, IL-6 and interferon (IFN)-γ were elevated in the right kidney biopsies from controls versus treated animals (Fig. 6a). Levels of IL-1β, tumour necrosis factor (TNF)-α, IL-8, MCP-1 and active transforming growth factor (TGF)-β were more variable between the two groups (Fig. 6a). Interestingly, protein levels of IL-2 were higher in the treated animals versus controls (Fig. 6a).

We also measured the proinflammatory mediators in kidneys harvested 3 or 6 months after AKI from the same two pairs (A1/C1 and A2/C2; Fig. 6b). RANTES, IL-6, IFN-γ, IL-1β, TNF-α and IL-2 were again elevated in right kidneys from control monkeys when compared with their left kidneys or to normal kidneys removed from four naive animals (Fig. 6b). IL-18 was elevated in both the right and left control kidneys. In contrast, all these mediators were minimally elevated in the right versus left kidneys of the mAb107-treated animals or when compared with the naive kidneys (Fig. 6b). No detectable changes in active TGF-β were observed in all cases (Fig. 6b).

Comparison of levels of the proinflammatory molecules in sera taken before, 1 day and 3 months after AKI showed elevated levels of RANTES, IL-18, IL-6, IFN-γ, IL-1β, TNF-α and IL-2 in control versus treated animals 3 months post injury (Supplementary Fig. 1). This probably reflects ongoing systemic inflammation in the control animal, a response known to be associated with progressive kidney failure^1^. No clear differences in MCP-1, IL-8 or TGF-β were observed at this time.

## Discussion

Preclinical studies have rarely examined the effects of prophylactic therapeutic interventions on long-term kidney function and structure following AKI. The present study, conducted in a reproducible CKD model in non-human primates, clearly shows that early inactivation of CD11b/CD18 by mAb107 interrupts the otherwise irreversible course of progressive kidney failure triggered by severe I/R AKI. This protective effect is observed even at 3, 6 or 9 months after severe AKI. Although mAb107 is a mouse IgG1, there was no evidence of enhanced leukocyte adhesion or CD11b/CD18 activation in treated monkeys, as judged by normal numbers of circulating leukocytes and lack of mAb24 binding to PMNs. This may be related to the fact that FcγR-mediated leukocyte adhesion requires a functional CD11b/CD18 (refs 17, 33). The present study also shows that severe AKI per se can progress to CKD in otherwise healthy non-human primates, seemingly resolving the existing controversy as to whether progression is caused by coexisting morbidities rather than by AKI per se^5^,^6^.

Ischaemic injury targets renal tubular epithelial cells, peritubular capillary endothelium and resident mononuclear phagocytes. The resulting lethal or sublethal cell damage triggers a local inflammatory response that involves a multitude of damage-associated molecular patterns, chemokines, cytokines, complement and coagulation factors. Attenuated vascular relaxation, swelling of ischaemic endothelial cells, capillary leak and adhesion of trapped leukocytes to each other, to the endothelium and to platelets inside capillaries prolong microvascular no-reflow even after vascular occlusion is released, which further aggravates ischaemic injury^30^,^31^,^34^. Prolonged no-reflow is also known to increase risk of primary kidney allograft dysfunction^35^.

Prophylactic treatment with mAb107 resulted in three early changes in the renal response to otherwise irreversible I/R AKI, which probably underlie its marked effect in mitigating progressive kidney fibrosis and failure. First, an immediate response to mAb107 treatment, visible by the naked eye (Fig. 2a and Supplementary Movies), was improved microvascular perfusion after vascular clamp release. The underlying mechanism is likely to be related to inhibition of CD11b-mediated leukocyte adhesion-dependent damage to peritubular capillary endothelium, as well as inhibition of formation of intracapillary PMN–platelet and PMN–PMN aggregates.

A second early response to mAb107 is stabilization of CD11b/CD18 in the inactive conformation. In studies of attempted blocking of other integrins, the inadvertent switch of the integrin to the active proadhesive state by ligand-mimetic small molecules or by allosteric monoclonal antibody inhibitors has been linked to serious adverse outcomes in treated patients^36^ and to failure of oral forms of the small molecule orthosteric inhibitors in clinical trials^28^. Previous short-term studies in rodent models of I/R AKI using monoclonal antibodies directed against CD18, ICAM-1 (a CD11b ligand) or CD11b yielded conflicting results^31^,^37^,^38^,^39^,^40^. This may be due in part to lack of specificity of anti-CD18 monoclonal antibodies (CD18 is shared by three other β2 integrins beside CD11b), activation of integrin signalling by the allosteric monoclonal antibody inhibitors and/or by the capacity of some anti-CD18 or anti-CD11b antibodies to synergize with other proinflammatory receptors^41^,^42^, potentially enhancing injury. In addition, simultaneous inhibition of multiple CD11b ligands rather than a single one (for example, ICAM-1) might have been necessary for an effective protective response.

A third early response to mAb107 therapy was a dramatic reduction in the pro-inflammatory proteins RANTES, IL-18, C3, IL-6 and IFN-γ in the ischaemic kidney biopsy, in association with significantly reduced numbers of CD45^+^ and CD11b^+^ leukocytes, and detectable improvement in renal histopathology in the right kidney biopsies, which was not reflected, however, in the simultaneous measurements of BUN/Cr. Levels of RANTES, IL-18, C3, IL-6 and IFN-γ remained low in the same right kidneys harvested from treated animals several months after the initial injury. RANTES, IL-18, complement, IL-6 and IFN-γ are produced by ischaemic parenchymal kidney cells and/or infiltrating NK cells and neutrophils^43^,^44^. Mouse knockout of RANTES^45^, IL-18 (ref. 46), complement^47^, IL-6 (ref. 44) or depletion of NK cells (an important IFN-γ source) partially protected kidney function 1–7 days post-I/R AKI. Simultaneous early reduction of all five molecules by the mAb107 indicates that intrinsic and/or the infiltrating leukocytes play a major role in their production/secretion. Whether this reduction results from inhibition of CD11b-mediated phagocytosis^26^, and/or Fcγ IIa/III and TLR4 signalling^24^,^25^, requires further investigation.

Levels of IL-1β, TNF-α, IL-8 and MCP-1 in treated right kidneys 1–2 days after I/R AKI were variable and did not correlate with long-term right kidney functional or structural improvement. This is consistent with the variable outcomes seen in rodent models of AKI, where double knockout of IL-1R and TNF-R1 did not protect against ischaemic AKI in mice^48^ or was somewhat protective in double knockout of IL-8 and MCP-1 in short-term studies^49^,^50^.

Interestingly, levels of IL-2, which is mainly produced by activated CD4^+^ T cells and dendritic cells^51^, were increased in mAb107-treated right kidneys 1–2 days after injury, but returned to near baseline in the harvested right kidneys. IL-2 plays an important role in expansion and/or function of CD4^+^CD25^+^ regulatory T cells^52^, which are known to attenuate I/R AKI in rodents^53^,^54^. To what extent the early rise in IL-2 in the right kidneys contributes to alleviation of progressive fibrosis later on remains to be clarified. Mice deficient in TGF-β1 are more prone to I/R AKI^55^. However, we did not observe changes in active TGF-β1 protein in kidneys or sera from treated or control animals, either shortly after injury or several months later.

The present data suggest that early targeting of CD11b/CD18 using the novel orthosteric inhibitor mAb107 may provide effective prophylaxis by mitigating progression of AKI to CKD. Future experiments will be necessary to determine the optimal therapeutic window for delivering mAb107 in our CKD model. However, prophylactic use of mAb107 as administered in the current protocol may already exert a therapeutic benefit in a number of clinical settings associated with development of AKI. These may include high-risk cardiac surgery (for example, elective coronary artery bypass grafting) or kidney transplantation. Ischaemic AKI is a common complication of cardiac surgery, especially in high-risk patients with renal hypoperfusion^56^, and carries an increased risk of progression to CKD and long-term mortality^57^,^58^. Ischaemic AKI after kidney transplantation is also a major cause of delayed graft function^49^, which increases the risk of long-term allograft failure^59^. In the latter setting, therapeutic infusion of mAb107 in kidney donor and recipient may be beneficial. Alternatively, adding mAb107 to the perfusion fluid during machine preservation^60^ may reduce postimplantation ischaemic injury in renal transplant patients. The present findings also suggest that mAb107 given as an adjunct to thrombolytic therapy or percutaneous coronary intervention may also be useful in prophylactic treatment of I/R injury to other organs such as the heart^61^.

## Methods

### Monoclonal antibody preparation

mAb107 was generated in mice immunized with human αA domain from integrin CD11b/CD18 (ref. 26). Alignment of human and cynomolgus αA domains showed 86% amino acid identity, but 100% identity in the αA residues contacting mAb107 that were previously defined in the crystal structure of the CD11b αA/107 Fab complex^27^. Purification of monoclonal antibody for *in vivo* use was done as described by Horenstein *et al*.^62^. Briefly, monoclonal antibody-containing supernatants harvested from hybridoma cultures were clarified and concentrated by circulating it through a hollow fibre membrane cartridge. The monoclonal antibody was purified by successive steps on Protein A affinity chromatography, hydroxyapatite charge exchange chromatography and size-exclusion chromatography. Endotoxin was removed using Detoxi-Gel (Pierce, Rockford, IL) and its level in the final product measured with Limulus Amebocyte Lysate gel-clot test. Monoclonal antibody concentration was determined by ultraviolet absorbance and purity assessed following SDS–PAGE and Coomassie staining.

### Animal procurement and procedures

Pathogen-free cynomolgus (macaque) male monkeys were bought from Charles River Laboratories (Wilmington, MA), weighed 2.6–4 kg and were ∼2–3 years old. They were housed in wire-mesh cages in air-conditioned, humidity- and temperature-controlled rooms with a 12 h light–dark cycle until use. Animals were fed LabDiet 5038 and tap water *ad libitum*.

All procedures were approved by the Institutional Animal Care and Use Committee of Massachusetts General Hospital. Anticoagulated blood was obtained from the saphenous vein. In preparation for surgery, monkeys were fasted overnight and sedated with intramuscular ketamine (3.0–5.0 mg kg^−1^) plus Dexdomitor (0.03–0.06 mg kg^−1^) and intubated. Atropine was given intramuscularly at 0.05 mg kg^−1^. Anaesthesia was maintained by administration of Isoflurane 1–3% inhalant, with intermittent administration of intravenous ketamine as needed. Vital signs and body temperature were continuously monitored. An intravenous infusion of normal saline containing 40 mg cefazoline was started using a 24 angiocatheter. Unilateral right kidney ischaemia was induced by a 2 h hilar cross-clamping. The contralateral left ureter was ligated after release of the vascular clamp. Five animals were treated with mAb107 (4–12 mg kg^−1^), two with the irrelevant isotype match mAbX63 and three with normal saline. The monoclonal antibody doses used were predicted to achieve blood levels of the mAb107 7–12-fold greater than the half-maximal phagocytosis-inhibitory concentration (*K*_i_∼15 μg ml^−1^)^26^ and were based on an estimated circulating blood volume of ∼65 ml kg^−1^ of body weight for cynomolgus monkeys. Three to 9 months post injury, the left ureter was surgically exposed and religated 2 days before planned euthanasia and the right kidney function was measured 1 and 2 days after the left ureter ligation. Open right kidney wedge biopsies were performed 1 and 2 days, and 1 month after reperfusion. The right kidney was exposed following a midline abdominal incision and a 2 × 4 mm incision was made with the M75 blade, extending ∼1–1/2 mm into the cortex. After assuring haemostasis, the wound was closed, the animal awakened, stable vital signs assured and then animal was returned to the housing area. Kidney sections were either snap frozen in liquid nitrogen or placed in 4% formaldehyde and embedded in paraffin. Animals were euthanized at the indicated times by intravenous administration of euthasol, 0.33 mg kg^−1^ (1 ml contains 390 mg of pentobarbital and 50 mg phenytoin). Both harvested kidneys were photographed and submitted for analyses.

### Measurement of non-reflow

A videotape of the surgical procedure was used to measure the time taken for the ischaemic dark purple-coloured right kidney to change colour back to red after release of the right vascular clamp.

### Quantification of peripheral blood leukocytes

Haematocrit, total white blood cells, neutrophils, monocytes, lymphocytes and platelets were quantified in heparinized blood using HemaTrue hematology Analyzer (Heska, Loveland, Colorado, USA) as described by the manufacturer.

### Biochemical determinations

Blood samples were allowed to clot and centrifuged at high speed for 30 min. Serum was stored at −80 °C until use. BUN and Cr were measured in duplicates in 96-well plates using a colorimetric method. Serum levels of the mouse monoclonal antibodies were measured using eBioscience mouse IgG total ELISA kit (Affymetrix, San Diego, CA) as described by the manufacturer.

### Proinflammatory mediators in kidney and serum

Collected kidney biopsy samples (25 mg each) were placed into 2 ml screw-top eppendorf tubes containing ice-cold 0.2 ml PBS and protease cocktail inhibitor (Roche Life Science, Indianapolis, IN). Next, a 5 mm stainless-steel bead was added to each tube and placed into a TissueLyser II machine (Qiagen, Waltham, MA) with an oscillation frequency of 30 Hz for 2 min. The levels of three chemokines (RANTES/CCL5, IL-8/CXCL8 and MCP-1/CCL2), seven cytokines (IL-1β, IL-2, IL-6, IL-18, TNF-α, IFN-γ and TGF-β1) and one complement protein (C3) in 0.050 ml lysed kidney extract or serum were assessed using magnetic bead kits run on a Luminex 200 system (EMD Millipore, Billerica, MA). Serum and kidney samples were tested undiluted, except samples undergoing C3 analysis, which were diluted 1:40,000 in assay buffer.

### Integrin activation and monoclonal antibody binding studies

PMN were purified from EDTA-anticoagulated monkey blood by fractionation over lympholyte-poly solution (CEDARLANE, Burlington, ON) as described by the manufacturer. Naive monkey PMN (1 × 10^6^ cells) in 100 μl Hank's balance salt solution containing 0.1% globulin-free BSA and 1 mM each of CaCl_2_ and MgCl_2_ (Hank's balance salt solution-BSA^++^) were treated with f-met-leu-phe (5 × 10^−7^ M final concentration) for 10 min at room temperature. Unlabelled Fab107 (75 μg ml^−1^) was added to one-half of the replicate tubes for an additional 30 min at 37 °C, followed by addition of mouse mAb24 IgG (20 μg ml^−1^) to all tubes and incubation for an extra 30 min at 37 °C. Cells were washed once, stained with anti-mouse Fc-specific fluorophore-labelled antibody (for 30 min at 0 °C), washed again, fixed with 1% formaldehyde in PBS and analysed using a FACS-Calibur or LSRFortessa flow cytometer (BD Biosciences, San Jose, CA). The FlowJo software was used to calculate mean fluorescence intensity. For detection of *in vivo* binding of mouse monoclonal antibody to monkey PMN, 1.4 μg of fluorescein isothiocyanate-anti-mouse Fc-specific monoclonal antibody (Jackson ImmunoResearch, West Grove, PA) was added to purified PMN and the mixture incubated for 20 min on ice. Cells were then washed once, fixed and analysed by flow cytometry.

### Histology and immunohistochemistry

Routine histology was performed on 6 μm kidney sections of formalin-fixed and paraffin-embedded tissue, and stained for Periodic Acid Schiff. Trichrome-stained slides used the Toulouse Latrec 1 Step Trichrome Stain Kit (Biocare Medical, Concord Park, CA). Immunohistochemistry (IHC) was performed as described by Farris *et al*.^63^. Rabbit polyclonal anti-CD45 antibody ab10588 and goat polyclonal anti-CD11b antibody ab62817 (both from Abcam, Cambridge, MA) were used at 1/100 dilution^64^. Epithelial cell necrosis was quantified in ten random fields at × 20 magnification. Macrophages were identified with anti-CD68 mAbED1 (Abcam). Cell count was expressed as mean+s.d. of all 20 random fields examined (at × 200 magnification). Fibrosis was expressed as the percent area that is Trichrome Stain-positive in ten fields at × 20 magnification. For collagen III IHC, antigen retrieval was performed using the Borg Decloaker (Biocare Medical). Polyclonal rabbit anti-human collagen III (LS-B693; Lifespan Biosciences, Seattle, WA) was used at a dilution of 1:400. For collagen III morphometry, stained sections were scanned with an Aperio Scan-Scope CS (Aperio Technologies, Inc., Vista, CA) and analysed using the ImageScope Positive Pixel Count algorithm (Aperio Technologies, Inc.). The Aperio Scanscope allowed scanning and quantification of the whole slide using a × 20 objective lens with a numerical aperture of 0.75 coupled with a doubler objective to achieve a scan of whole slides at × 40 magnification. The default parameters of the Positive Pixel Count (hue of 0.1 and width of 0.5) detected collagen III IHC adequately. Collagen deposition was expressed as percent of pixels positive for anti-collagen antibody.

### Statistical calculations

Based on the dramatic differences in the initial data using a control and a treated animal, if only 20% of right kidneys survive in the control group (s.d.=15) and 65% in the treated group (s.d.=15), a significance level of 0.02 is achieved using a total sample size of four animals in each group, yielding ≥95% statistical power^65^. For sample comparisons, data were analysed by the unpaired two-tailed Student *t*-test or by the Mann–Whitney *U*-test. *P*-values <5% were considered significant.

### Data availability

The data that support the findings of this study are available from the corresponding author upon reasonable request.

## Additional information

**How to cite this article:** Dehnadi, A. *et al*. Prophylactic orthosteric inhibition of leukocyte integrin CD11b/CD18 prevents long-term fibrotic kidney failure in cynomolgus monkeys. *Nat. Commun.* **8,** 13899 doi: 10.1038/ncomms13899 (2017).

**Publisher's note**: Springer Nature remains neutral with regard to jurisdictional claims in published maps and institutional affiliations.

## Supplementary Material

Supplementary Information Supplementary Figure.

Supplementary Movie 1 Representative video showing the time taken for ischemic right kidney form a control monkey (C1) to regain its pink colour following release of the vascular clamp. The video starts immediately after vascular clamp release.

Supplementary Movie 2 Representative video showing the time taken for ischemic right kidney form a mAb107-treated monkey (A1) to regain its pink colour following release of the vascular clamp. The video starts immediately after vascular clamp release.
