# Supplementary material for: Prophylactic orthosteric inhibition of leukocyte integrin CD11b/CD18 prevents long-term fibrotic kidney failure in cynomolgus monkeys
Source: Nat Commun. 2017 Jan 10;8:13899. doi: 10.1038/ncomms13899 (PMC5234083; doi:10.1038/ncomms13899)
Supplement: Supplementary Information — Supplementary Figure. [file ncomms13899-s1.pdf]

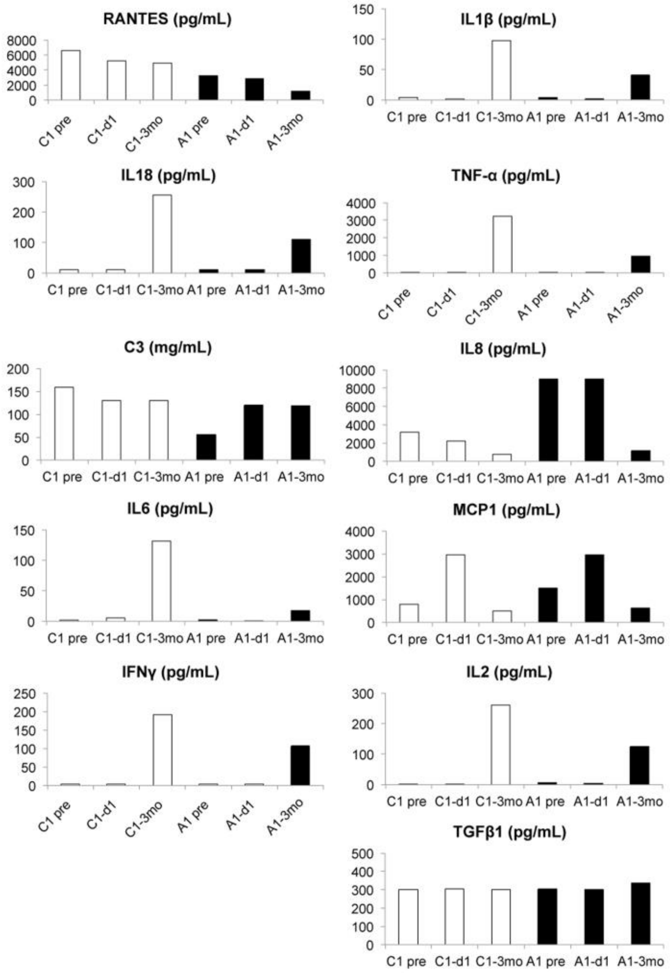


Supplemental Fig.1

**Supplementary Figure 1**. Serum levels of proinflammatory mediators. Histograms showing levels of proinflammatory mediators in sera obtained prior to surgery (pre), on d1 and at 3 months after ischemic injury in a control (C1) and treated (A1) monkeys. See text for details.
